# CO_2_ reactivity as a biomarker of exposure-based therapy non-response: study protocol

**DOI:** 10.1186/s12888-022-04478-x

**Published:** 2022-12-27

**Authors:** Jasper A. J. Smits, Marie-H. Monfils, Michael W. Otto, Michael J. Telch, Jason Shumake, Justin S. Feinstein, Sahib S. Khalsa, Adam R. Cobb, E. Marie Parsons, Laura J. Long, Bryan McSpadden, David Johnson, Alma Greenberg, Exposure Therapy Consortium

**Affiliations:** 1 Department of Psychology and Institute for Mental Health Research, University of Texas at Austin, 1 University Station, Austin, TX 78712 USA; 2 Department of Psychological and Brain Sciences, Boston University, 900 Commonwealth Avenue, Floor 2, Boston, MA 02215 USA; 3 3The Laureate Institute for Brain Research, 6655 South Yale Ave., Tulsa, Oklahoma 74136 USA; 4 Department of Psychiatry and Behavioral Sciences, Medical University of South Carolina and Ralph H. Johnson VAHCS, 67 President Street MSC 862, Charleston, SC 29425 USA

**Keywords:** Panic disorder, Social anxiety disorder, Obsessive–compulsive disorder, Generalized anxiety disorder, Posttraumatic stress disorder, Exposure therapy, Biomarker, Orexin, CO_2_ challenge, Clinical trial

## Abstract

**Background:**

Exposure-based therapy is an effective first-line treatment for anxiety-, obsessive–compulsive, and trauma- and stressor-related disorders; however, many patients do not improve, resulting in prolonged suffering and poorly used resources. Basic research on fear extinction may inform the development of a biomarker for the selection of exposure-based therapy. Growing evidence links orexin system activity to deficits in fear extinction and we have demonstrated that reactivity to an inhaled carbon dioxide (CO_2_) challenge—a safe, affordable, and easy-to-implement procedure—can serve as a proxy for orexin system activity and predicts fear extinction deficits in rodents. Building upon this basic research, the goal for the proposed study is to validate CO_2_ reactivity as a biomarker of exposure-based therapy non-response.

**Methods:**

We will assess CO_2_ reactivity in 600 adults meeting criteria for one or more fear- or anxiety-related disorders prior to providing open exposure-based therapy. By incorporating CO_2_ reactivity into a multivariate model predicting treatment non-response that also includes reactivity to hyperventilation as well as a number of related predictor variables, we will establish the mechanistic specificity and the additive predictive utility of the potential CO_2_ reactivity biomarker. By developing models independently within two study sites (University of Texas at Austin and Boston University) and predicting the other site’s data, we will validate that the results are likely to generalize to future clinical samples.

**Discussion:**

Representing a necessary stage in translating basic research, this investigation addresses an important public health issue by testing an accessible clinical assessment strategy that may lead to a more effective treatment selection (personalized medicine) for patients with anxiety- and fear-related disorders, and enhanced understanding of the mechanisms governing exposure-based therapy.

**Trial registration:**

ClinicalTrials.gov Identifier: NCT05467683 (20/07/2022).

## Background

Anxiety-, obsessive–compulsive and trauma- and stressor-related disorders are prevalent and costly mental health disorders [1–4]. Exposure-based therapy (EBT) has demonstrated efficacy [5] and is a recommended first-line treatment for these disorders [6–10]. As the utilization of EBT has strengthened [11], it becomes more important to identify biomarkers of EBT non-response [12]—an outcome observed in a significant subset of patients [13–17]. Indeed, making available easy-to-implement screening tools to aid clinicians and patients in deciding whether to initiate EBT can reduce “unnecessary” prolonged suffering and treatment burden/costs, as well as increase the availability of EBT therapists for patients most likely to respond to this treatment.

Although there have been efforts to identify predictors of EBT non-response, these have been largely post-hoc or based on the secondary analyses of efficacy studies [18–30], and consequently they have been handicapped by low statistical power and replication failure. As we detail next, a fruitful approach to identifying “clinically meaningful” biomarkers of EBT non-response is to (1) focus on variables that are (theoretically) related to core mechanisms of action of EBT, especially when such variables can be readily assessed in clinical practice; and (2) employ a methodological approach that facilitates the goal of reproducibility.

### Theory-informed biomarkers

EBT was derived from models of extinction learning [31]. The focus on developing safety memories/fear extinction is at the heart of EBT for anxiety-, obsessive–compulsive and trauma- and stressor-related disorders [31, 32]. Through guided experiences involving the presentation of feared cues, clinicians help patients undo the maladaptive anxiogenic beliefs, vigilance to harm, and avoidance that characterize these disorders. Importantly, consistent with the hypothesis that fear extinction is a core mechanism of EBT [31, 33, 34], a growing body of literature links subjective and neural indices of fear extinction to EBT outcomes [35–40]. Hence, identifying variables that predict fear extinction can aid clinical decision-making with respect to the selection of EBT [41].

Orexins are neuropeptides produced by neurons localized exclusively in the hypothalamus [42, 43]. Orexin-expressing neurons have extensive projections within the CNS and, as such, orexins play a role in several important functions, including sleep, feeding, and anxiety [44–46]. Experimental evidence suggests an important role of the orexin system in fear extinction [47–51]. Indeed, greater activation of orexin neurons in the medial hypothalamus is associated with poor fear extinction [52], blocking the (orexin/)hypocretin-1 (Hcrtr-1) receptor with the antagonist SB334867 facilitates fear extinction [48, 50], and administering Hcrtr-1 impairs fear extinction [48]. Furthermore, antagonism of orexin receptors increases the recruitment of basolateral amygdala (BLA) neurons that project to the infralimbic cortex (IL) during extinction [50]. Those very same neurons (the IL-projecting BLA neurons) are found to be active during fear extinction [53], underscoring the observation that orexin activation in the lateral hypothalamus (LH) accounts for individual differences in fear extinction [47]. Translation of these rodent findings to the clinic is aided by a study involving patients with panic disorder that has linked the Hcrtr-1 rs2271933 genotype to poor response to EBT, implicating orexin activity as a marker of EBT non-response [54].

The relation between orexin cell activity and the aforementioned processes has been well established, particularly in non-human animals, where the cell activity can be directly quantified [49]. In humans, this relation has been extended to patients in which microdialysis could be performed [55]; however, most studies conducted in humans have quantified orexin either in plasma, saliva, or cerebrospinal fluid (CSF). Whereas orexin quantified from CSF appears to correlate with orexin sampled from the LH, orexin quantified from plasma or saliva does not correlate with orexin in the CSF, suggesting that modulations occurring in line with the findings established in rodents are specific to the central nervous system (CNS). Fluctuations in plasma orexin A are also not differentially modulated by orexin receptor gene polymorphisms [56]. Furthermore, orexin A is present in the blood in low amounts, and its levels do not follow autonomic or neuroendocrine circadian rhythms [57], suggesting that evaluations of orexin quantifications outside of CNS should be interpreted very cautiously.

Given these limitations, carbon dioxide (CO_2_) reactivity emerges as perhaps the best non-intrusive index of orexin activity. Indeed, prepro-orexin knockout mice evidence reduced respiratory reactivity to hypercarbic gas exposure (CO_2_ challenge) and pre-CO_2_ challenge injection of the Hcrtr-1 receptor antagonist SB334867 reduces respiratory reactivity in wild-type mice [58]. Similarly, blocking the Hcrtr-1 receptor with the administration of Hcrtr-1 receptor antagonists (e.g., SB334867, JNJ-61393215) reduces fear and anxious responding to CO_2_ challenge both in rats and in humans [59, 60]. Collectively, these findings show that the orexin system (and particularly the Hcrtr-1 receptor) mediates hypercapnia-induced fear and sympathetic drive and, by extension, suggest that CO_2_ reactivity can serve as a proxy to index orexin activity [51, 59, 60].

The CO_2_ challenge is a safe and well-tolerated procedure that involves the inhalation of CO_2_-enriched air at various concentrations (e.g., 5%, 7%, 15%, 20%, or 35%)[61]. Important for studies translating non-human animal work is the observation that CO_2_ challenge fear reactivity (i.e., behavior in rats, subjective ratings and avoidance in humans) as well as respiratory and cardiovascular reactivity are comparable across species [62]. Stemming from physiological systems that are distinguishable from those subserving general trait anxiety and lie on a continuum with the extreme being panic [63], the CO_2_-driven subjective and behavioral responses in humans are dose-dependent [64] and elevated relative to healthy control subjects among individuals with panic disorder (PD) [65–67], social anxiety disorder (SAD) [68, 69], generalized anxiety disorder (GAD) [70, 71], obsessive–compulsive disorder (OCD) [72], and posttraumatic stress disorder (PTSD) [73, 74], while also demonstrating adequate intra-individual variability within each of these diagnostic categories.

### Optimizing the methodological approach to validating putative biomarkers

We conceptualize reactivity to hyperventilation as a control biomarker because voluntary hyperventilation (VH): (1) reliably induces affective reactivity in those with anxiety and related disorders [75–78]; (2) shares method variance with the CO_2_ challenge (i.e., uses the respiratory system and an identical strategy for indexing subjective reactivity); (3) reactivity is predicted (as is CO_2_ reactivity) by psychological variables relevant to fears of somatic sensations [76–78]; and (4) has the opposite effect to CO_2_ challenge on pH and thus orexin cell firing in the LH: acidification (i.e., decrease in pH resulting from an increase in CO_2_) increases intrinsic excitability, whereas alkalinization (i.e., increase in pH resulting from a decrease in CO_2_) depresses it [79–81], and thus is not considered a marker of orexin activity. Hence, VH allows for disentanglement of reactivity due to fears of respiratory somatic sensations and to reactivity more directly reflecting orexin activity (via CO_2_ challenge).

We utilize converging self-report, behavioral, and physiologic methods to index CO_2_ reactivity, including: (1) self-reported peak anxiety reactivity and habituation/sensitization in anxiety, (2) behavioral latency between inhalations (avoidance/escape), and (3) physiologic reactivity during recovery (end-tidal CO_2_). All three indices are available from a single-session CO_2_ challenge; hence, we retain the efficiency of our procedures while providing a comprehensive set of subjective and objective indices.

CO_2_ reactivity can also be influenced by a number of psychological factors. We have selected control variables relevant to CO_2_ reactivity that are not related to orexin activity, including: (1) anxiety sensitivity (AS; the tendency to perceive that anxiety-related symptoms and sensations have catastrophic consequences [82]), which operates as a transdiagnostic risk factor for the maintenance of anxiety pathology [83] and a reliable predictor of reactivity to interoceptive challenges, including CO_2_ [84, 85] and VH [78, 86]; (2) distress intolerance (DI); notably, although AS can be conceptualized as a measure of distress intolerance (DI), there is variability in prediction and reactivity with alternative measures of DI [87–89]); (3) experiential avoidance (EA) [90]; (4) Intolerance of uncertainty (IU) [91, 92], a putative transdiagnostic risk factor for the maintenance of anxiety and related disorders and EBT non-response [93–99]; and finally, (5) a behavioral index of subtle avoidance behaviors that may interfere with extinction [100]. In sum, AS, DI, EA, IU, and related avoidance tendencies all share variance with CO_2_ and VH reactivity, but like VH, should be a poor marker of orexin activity. Finally, to demonstrate that the CO_2_ derived biomarker offers incremental predictive utility, we will also include standard, albeit unreliable, clinical and demographic predictors of EBT non-response [18–30].

### Adopting a stage model approach for biomarker research

The identification of biomarkers for psychosocial treatment response has been marked by difficulties in replication. For this reason, we believe that establishing reliability for a biomarker is more important than establishing the treatment specificity of that marker in the first stage of biomarker validation. This is analogous to the stage approach to treatment development; we are starting with a Stage 1 (open) evaluation of biomarker adequacy. Accordingly, our design maximizes reliability by including a replication sample in the design to ensure consistency of prediction for the putative biomarker. Consistent with a Stage 1 approach [101], our design does not include a test to ensure that the non-response predicted by the biomarker is specific to EBT vs. some alternative treatment. Indeed, the selection of which treatment should serve as an alternative treatment is a premature decision for two reasons: (1) we have not yet determined the level at which CO_2_ reactivity becomes predictive of non-response (hence, it is unclear what cut-off can be used to stratify based on biomarker status [+ or -]); and (2) it is unclear at this time what intervention can be used effectively either because it does not rely on orexin function or because it can manipulate the orexin system.

### Sample size and data analytic approach

Simulation work has shown that large sample sizes are required to develop accurate predictive models of treatment response [102, 103]. Our own statistical simulation shows that given the two-site, split-half validation design, we require 300 participants per site—at least 300 to develop the model and another 300 to adequately validate it in this Stage 1 model. The reality is that underpowered studies likely detect only the largest effects, and multivariate models based on them yield unstable predictions that overfit the sample data and perform poorly out-of-sample [104, 105]. As pointed out by Kessler and colleagues, “The inevitable conclusion is that samples much larger than those in existing mental disorder randomized clinical trials are required to develop useful personalized treatment recommendations” [106]. The overarching message of the statistical learning literature and our own simulations used to develop this proposal is “go big or go home”.

Our primary objective is to build a model predicting EBT non-response using a multivariate combination of candidate predictors. One set of predictors constitutes what we consider our “control” model; these include “traditional” prognostic factors and psychological variables (see Table 1). The addition of a second set of predictors, corresponding to our proposed measures of reactivity to CO_2_ and VH, constitutes our “experimental” model. We have two key hypotheses that we aim to test: (1) the experimental model will outperform the control model when predicting never-before-seen data, establishing the additive predictive value of assessing reactivity measures prior to initiating treatment; and (2) when predicting never-before-seen data, a model that excludes VH reactivity measures will outperform a model that excludes CO_2_ reactivity measures, establishing that the additive predictive value is specific to CO_2_ reactivity.

**Table 1:** Schedule of assessments

Measurement Domain	Screen	Biomarker Visit	Treatment (Weeks 1–12)	Follow-up (Weeks 13, 24)
***Primary Outcome Measures***				
*Clinician Rated*: CGI-S/CGI-I			X	X
*Patient Rated*: OASIS	X		X	X
***Secondary Outcome Measures***				
*Patient Rated*:^a^			X	X
PD—PDSS-SR				
SAD—SPIN				
GAD—GAD-7				
OCD—DOCS				
PTSD—PCL-5				
Depression—PHQ-9				
***Screening***				
Internet prescreen, Demographics, SCID-5, CSSR-S	X			
***CO***_***2***_*** and VH Reactivity Measures***				
Peak anxiety, Habituation/Sensitization, Latency, End tidal CO_2_		X		
***Theoretically-Relevant Competing*** ***Predictor Variables***				
ASI-3, DTI, BEAQ, IUS, SBAF		X		
***Control Predictor Variables***				
Sex assigned at birth, Gender identity, Diagnosis, Comorbidity symptom severity	X	X		
***Treatment Integrity and Acceptance***				
CEQ^b^, Attendance			X	

^a^For these measures patients will only complete the scale thatassesses symptom severity regarding their primary DSM-5 disorder (all participants will complete the PHQ-9)

^b^Only administered after the first treatment session

*PD* Panic disorder, *SAD* Social anxiety disorder, *GAD* Generalized anxiety disorder, *OCD* Obsessive–compulsive disorder, *PTSD* Posttraumatic stress disorder, *SPIN* Social Phobia Inventory, *DOCS* Dimensional Obsessive Compulsive Scale, *PCL-5* PTSD Checklist for Diagnostic and Statistical Manual of Mental Disorders-Fifth Edition, *PDSS-SR* Panic Disorder Severity Scale-Self Report Version, *OASIS* Overall Anxiety Severity and Impairment Scale, *CGI* Clinical Global Impressions, *CGI-I* Clinical Global Impressions—Improvement, *CGI-S* Clinical Global Impressions—Severity, *SCID-5* Structured Clinical Interview for DSM-5, *C-SSRS* Columbia-Suicide Severity Rating Scale, *ASI-3* The Anxiety Sensitivity Index, *DTI* The Distress Tolerance Index, *IUQ* The Intolerance of Uncertainty Questionnaire, *BEAQ* The Brief Experiential Avoidance Questionnaire, *SBAF* The Safety Behavior Assessment Form, *CEQ* Credibility/Expectancy Questionnaire

Importantly, cross-validation is used during model development to prevent overfitting, and all statistical tests of model predictions will be performed using data collected from a completely independent site located in a different geographic region of the country. This ensures that our findings will not just be “statistically significant” in the sense of being improbable under a null hypothesis, but will demonstrate real-world predictive utility in two respects: (1) the biomarkers must out-perform other measures and (2) they must predict new data, showing that predictions based on one clinical sample will generalize to another. We have powered our proposed study to meet this higher standard. If we are successful, it is our further objective to translate our model predictions into a set of decision rules to facilitate their implementation in clinical practice.

## Methods/design

### Design

We aim to recruit a transdiagnostic sample (*N* = 300 at each of two collaborating sites [The University of Texas at Austin (UT) and Boston University (BU)]) presenting with one or more DSM-5 anxiety disorders, OCD, or PTSD. Eligible participants will complete 20% CO_2_ and VH challenges, as well as a comprehensive set of clinician-rated and patient-rated measures prior to starting open, transdiagnostic, EBT. Assessment of non-response will occur weekly during treatment, at 1-week posttreatment (i.e., primary endpoint), and at 3-month follow-up. This study has been registered with ClinicalTrials.gov (NCT05467683; 20/07/2022). The UT Institutional Review Board has approved the study protocol for both participating sites (STUDY00001631).

### Participants

The transdiagnostic sample will consist of 600 participants. Inclusion criteria include: (1) a primary DSM-5 diagnosis of PD (with or without agoraphobia), SAD, GAD, OCD or PTSD; (2) a score of 8 or greater on the Overall Anxiety Severity and Impairment Scale (OASIS) [107]; (3) ages 18–70; (4) willingness and ability to provide informed consent and comply with the requirements of the study protocol; and (5) proficiency in English (because many assessment instruments have only been validated in English). Exclusion criteria include: (1) a lifetime history of bipolar or psychotic disorders; substance use disorders (other than nicotine) or eating disorder in the past 6 months; serious cognitive impairment; (2) active suicidal ideation with at least some intent to act with or without a specific plan (i.e., a rating of 4 for suicidal ideation on the Columbia-Suicide Severity Rating Scale; CSSRS) [108] or suicidal behaviors (actual attempt, interrupted attempt, aborted or self-interrupted attempt, or preparatory acts or behavior) within the past 6 months; (3) medical conditions contraindicating CO_2_ inhalation or VH (e.g., cardiac arrhythmia, cardiac failure, asthma, lung fibrosis, stage 2 high blood pressure, epilepsy, or stroke); (4) pregnancy or lactation; (5) ongoing psychotherapy directed toward the primary disorder; or (6) pharmacological treatment started within 8 weeks prior to the screen (participants “stable” on their medication regimen will be included and their medication status will be included as a variable in the model).

#### Recruitment

Participants will be recruited from our outpatient clinics specializing in the treatment of fear- and anxiety-related disorders, which helps ensure adequate flow for the proposed study. To complement the natural flow, we will advertise through numerous community organizations, social media platforms, and other internet-based referral sources.

#### Retention

We include an incentive-based approach that includes; (1) obtaining multiple methods for contacting participants; (2) offering flexibility in scheduling appointments; (3) personalized connections around scheduling; (4) providing reminders of appointments; and (5) weekly monitoring of recruitment and retention and quality control across sites. Also, assessment adherence and study completion will be aided by study compensation. Compensation is based on one biomarker assessment session, one baseline session, 12 weekly assessment sessions, 1 posttreatment and 1 follow-up assessment at the following levels: $70 for the biomarker assessment session, $30 for the baseline assessment, $10 for each weekly assessment ($120), and $30 for posttreatment and follow-up ($60), plus a reward of $20 for completing all assessments, for a total of $300 per participant.

### Procedures

#### Screening

An internet prescreen will be conducted for all potential participants. Persons who appear eligible will be invited to complete diagnostic screening. Participants will receive an informed consent form explaining the details of the study, potential benefits and risks of participation, and the procedures they will undergo if they choose to participate. If the individual provides informed consent, they will begin the psychiatric evaluation process, which will be conducted during an in-person visit with a clinician.

#### Biomarker visit

Prior to the first treatment visit, participants will complete two distinct respiratory challenges to assess the putative biomarker and relevant control variables.

##### CO_2_ challenge

The CO_2_ challenge comprises two 20-min trials: the 1^st^ trial involves 3 vital capacity (VC) breaths from a bag of compressed air and the 2^nd^ trial involves 3 VC breaths from a bag containing 20% CO_2_-enriched air (participants will be blind to the content of the inhalation mixture in the bag). Participants will first view a video recording that provides information about CO_2_ inhalation and modeling of the CO_2_ challenge procedures. The integrated system built by Hans Rudolph, Inc. and customized for this study includes a pulse oximeter (to assess heart rate and blood oxygen saturation; exploratory measures), a breathing mask with sensors (to measure tidal volume, respiratory rate, minute ventilation, end-tidal O_2_, and end-tidal CO_2_), a dial (for continuous ratings of anxiety) and a button to initiate inhalation. Prior to the first trial, participants will practice VC inhalation (exhaling completely and inhaling to maximum lung capacity). Participants will then be allotted 20 min to complete 3 VC inhalations at their own pace by using the button to initiate each inhalation. They will be asked to rate how much anxiety they feel, moment by moment, using the rating dial. The two trials—involving VC breaths of room air followed by 20% CO_2_-enriched air—are each preceded by a 2-min baseline period and followed by a 2-min recovery period.

##### VH challenge

The VH challenge will be administered within 30 min following the CO_2_ challenge. It comprises one 20-min trial involving 3 two-minute VH provocations (matching the CO_2_ challenge procedures). Consistent with recommendations for standardization [109], participants will view a video recording that explains hyperventilation and the challenge procedures and models proper VH (i.e., breathing in pace with pacing tones signaling inspiration and expiration to guide the rate of 18–24 breaths per minute) [109] and then complete a 15-s practice trial supervised by staff. Participants will be fitted with the same pulse oximeter (to assess heart rate) and breathing mask which will allow assessment and monitoring of tidal volume, respiratory rate, minute ventilation, end-tidal O_2_, and end-tidal CO_2_, and provide the staff with the necessary feedback to potentially modify breathing rate to ensure that the participant stays at the target end-tidal CO_2_ level (i.e., 20 mmHG) for 2 min [109]. They will rate anxiety using the dial continuously, and will be told that they have 20 min to complete three VH provocations at their own pace with a button press to initiate each trial, mirroring the CO_2_ challenge procedures.

#### Open exposure-based therapy

Transdiagnostic EBT will be delivered by experienced, license-eligible clinicians. To aid generalization to EBT delivered in clinical practice, the study clinician will develop a personalized assessment and treatment plan for each participant. Assessment algorithms will (1) guide the case formulation, which emphasizes threat appraisals as maintaining factors to be targeted during treatment; and (2) provide the data for tracking success and progress. The case formulation guides the clinician in the development of personalized exposure exercises, while tracking success and progress allows for updating of the case formulation and fine-tuning of the treatment plan. Consistent with contemporary models of EBT [31, 110], exposure practice aims to help patients relearn a sense of safety around feared cues. Hence, exposure exercises are planned to ensure violation of threat expectancies. In addition to ensuring sufficient activation of the “fear network” and a focus on repetition to provide disconfirmatory evidence, exposure practice will be planned and delivered keeping in mind that fear extinction tends to be context specific. Specifically, practice will occur across relevant contexts both within and outside the session (i.e., homework) and clinicians will guide participants in processing their exposure practice to facilitate consolidation of safety learning.

To achieve these ends, study clinicians will use a manual that describes these procedures for treatment planning and delivery. The manual “Personalized Exposure Therapy: A Person-Centered Transdiagnostic Approach [32]” includes clear guidance on the conceptual model of EBT, assessment planning and strategies, and separate chapters on the planning and delivery of in vivo, imaginal and interoceptive exposure practice, respectively. The treatment dose will be set at 12 one-hour sessions, delivered over the course of 12 weeks. The quality assurance protocol for treatment implementation involves requiring all clinicians to (1) complete a 6-h training workshop; and (2) attend weekly supervision meetings.

### Assessment

Table 1 provides an overview of assessment targets and measures by study phase.

#### Screening

The online questionnaire first asks potential participants to provide standard demographic information and to indicate whether they have experienced or been diagnosed with any of the psychiatric or medical exclusion criteria. Participants who do not endorse exclusion criteria will then be asked to complete the OASIS [107]. Participants who endorse experiencing anxiety-related symptoms and impairment will then complete the DSM-5-TR Self-Rated Level 1 Cross-Cutting Symptom Measure [111]. Based on their responses to this measure, participants will also complete relevant Level 2 or diagnosis-specific measures, which for the current study include the Severity Measure for Panic Disorder [112], Mobility Inventory (MI; alone) [113], Social Phobia Inventory (SPIN) [114], PROMIS Emotional Distress—Anxiety—Short Form [115], Dimensional Obsessive Compulsive Scale [DOCS; OCD] [116], and the PTSD Checklist for Diagnostic and Statistical Manual of Mental Disorders-Fifth Edition (PCL-5) [117] as well as measures of constructs that aid the case formulation (e.g., anxiety/fear cues, core threat appraisals, and safety behaviors). Participants that do not meet any exclusion criteria after this screening will be given the option to schedule an in-person screening visit.

The clinician assigned to the participant will complete the in-person screen visit, which includes administration of the Structured Clinical Interview for DSM-5 (SCID-5) [118] and the Columbia Suicide Severity Rating Scale (C-SSRS) [108].

#### Biomarker measures

For each VC inhalation, mean and peak reactivity will be assessed across different epochs (i.e., baseline, anticipation, peak response, and recovery). For the subjective anxiety index, difference scores will be calculated between the 1^st^ trial (bag of compressed air) and 2^nd^ trial (bag of CO_2_). The primary subjective measure of CO_2_ peak anxiety reactivity will be the participant’s peak real-time ratings of subjective anxiety (reported throughout the trials using the rating dial). The primary measure of the degree of habituation vs. sensitization will be the change in peak subjective anxiety from the 1^st^ to the 3^rd^ of the CO_2_ provocations. The primary behavioral measure will be avoidance responses (latency in seconds between inhalations 1–2, and 2–3). The primary physiological measure will be the difference in end-tidal CO_2_ during the resting baseline vs. recovery phase.

For each of the three VH provocations, mean and peak reactivity will also be assessed across epochs (i.e., baseline, anticipation, peak response, and recovery). For the subjective index, difference scores will be calculated between VH baseline (at rest) ratings and the trial (VH) ratings. The primary subjective measure of VH peak anxiety reactivity will be the participant’s peak real-time ratings of subjective anxiety (reported throughout the trials using the rating dial). The primary measure of the degree of habituation vs. sensitization will be the change in peak subjective anxiety from the 1^st^ to the 3^rd^ of the VH provocations. The primary behavioral measure will be avoidance responses (latency in seconds between VH provocations 1–2, and 2–3). The primary physiological index of VH reactivity will be the mean difference in end-tidal CO_2_ during the resting baseline vs recovery phase.

#### Theoretically-relevant and general competing predictor variables

Participants will complete the following self-report measures of constructs related to CO_2_ reactivity and/or expected to be predictive of EBT non-response: ASI-3 [119], Distress Tolerance Index (DTI) [88], Brief Experiential Avoidance Questionnaire (BEAQ) [120], IUS [91], and Safety Behavior Assessment Form (SBAF) [121]. Additional control predictor variables include sex assigned at birth, gender identity, number of diagnoses (s measured by the SCID-5), and comorbidity symptom severity (as measured by the DSM-5-TR Self-Rated Level 1 Cross-Cutting Symptom Measure).

#### Symptom severity

Prior to each treatment visit (Weeks 1–12), at posttreatment (Week 13) and at follow-up (week 24), an independent evaluator (IE; telehealth) will administer the Clinical Global Impressions (CGI) scales [122]. Participants will also complete the OASIS and the Patient Health Questionnaire (PHQ-9) as well as the symptom severity measure corresponding to their primary diagnosis (Panic Disorder Severity Scale-Self Report Version [PDSS-SR; PD] [123], SPIN [SAD] [114], GAD-7 [GAD] [124], DOCS [OCD] [116], or PCL-5 [PTSD] [117]) prior to the meeting with the IE. Every 3 weeks participants will also be asked to complete several treatment process measures that are not related to the primary study aim. IE training will involve completion of a 3-h workshop and reliable rating (> = 80%) of interviews with test subjects. IE’s will also complete quarterly ratings of test cases to prevent rater drift.

##### Definition of non-response

Participants will be classified as non-responders if their CGI—Improvement (CGI-I) score is 3 or above OR if their OASIS score has not improved by at least 4 points.

#### Treatment integrity and acceptance

Participants will complete the Credibility/Expectancy Questionnaire (CEQ) [125] which is a widely used 6-item measure that assesses treatment credibility and expectancy, after the first treatment session. Participant adherence to each intervention will be assessed as the number of total sessions attended.

### Data analysis

#### Rationale for statistical learning approach

Our primary goal of identifying mechanistic non-response indicators that can be readily assessed in clinical practice requires a machine learning approach that has (as its end product) a single model that any clinician can easily understand and adopt. Whereas there is evidence supporting the potential relevance of all our candidate predictors for individual bivariate relationships with non-response, we do not yet know how to optimally combine them into a single model to maximize prediction accuracy. This is especially true for the CO_2_/VH reactivity measures, which are multimodal, including physiology, behavior, and self-report. It is unlikely that all of these measures will provide a unique predictive signal; it is very likely that some may be redundant, and others may constitute noise. We therefore require a data-driven method of variable selection that reveals the best subset of predictors. A traditional approach would be to use a generalized linear model (GLM) and a stepwise selection algorithm, in which model terms are iteratively added or deleted and the model is repeatedly refit until some information criterion settles into a local optimum. Numerous weaknesses of such stepwise selection have been noted [126] including model selection bias, which can exaggerate the apparent strengths of relationships [127, 128].

The essential problem is that, with or without variable selection, an ordinary logistic regression is very likely to overfit the data [129] (meaning the model is trained to predict sample noise, which leads to inflated estimates of how well the model predicts the sample it was trained on, at the expense of generalizability when predicting other samples). Thus, a statistical learning technique is required that can handle the potential for highly correlated covariates and discourage overfitting. In developing a statistical learning approach for our objectives, we are mindful of the tradeoff between prediction accuracy and model interpretability that exists within the large umbrella of machine learning approaches. Generally speaking, the approaches with the best prediction performance (e.g., stacked ensembles that blend a diversity of machine learners) are the most successful in revealing comprehensible mechanisms. This has led us to select regularized regression, using the elastic net penalty, as our primary approach.

The chief advantage of the elastic net [130] is that it is still a GLM, and therefore its model output is as easy to understand and interpret as any GLM. Importantly, elastic net regression functions just as a GLM would in terms of handling covariates. For instance, if CO_2_ reactivity is entered into the model alongside other predictors, its estimated regression coefficient will reflect its incremental contribution to the prediction, controlling for all other covariates. The only difference is that the optimization procedure that fits the model, in addition to maximizing the likelihood of the observed data, works to minimize the size of the model coefficients, which is variously referred to in the literature as penalizing, regularizing, or shrinking the coefficients. The elastic net penalty is a mixture of “lasso” (L1) and “ridge” (L2) penalties. The lasso component favors sparsity by allowing the coefficients of the least influential covariates to be shrunk to 0, effectively selecting them out of the model entirely, while the ridge component favors inclusivity by enabling highly correlated variables to be shrunk together instead of arbitrarily picking one and discarding the rest. Importantly, in contrast to stepwise selection, variable selection is a by-product of coefficient shrinkage and the models are fit using all of the predictors. Cross-validation (tenfold) is used to tune the optimal combination and magnitude of penalties for a “just right” fit—flexible enough to capture real complexity, but constrained enough to avoid capturing noise—by choosing the amount of regularization that maximizes out-of-sample generalization instead of in-sample fit.

A potential disadvantage of the elastic net is that it does not provide an automatic search for potential nonlinear relationships or higher-order interactions among predictors, which other machine learning algorithms (e.g., random forests) would supply. However, in our publications and experience building machine learning ensembles to predict mental health outcomes [131], the elastic net has been the major workhorse of these ensembles, and the gains from adding other machine learners have been minimal at best (likely because the accurate capture of nonlinear, high-order interactions will require enormous sample sizes much larger than the ones we have worked with to date [132]). However, to assess how much loss of predictive accuracy an elastic-net may entail relative to more black-box approaches, we will also use superlearning/stacked ensembles, which have been touted as an optimal way to discover treatment rules that maximize response outcomes [133].

#### Model development and validation strategy

We will maintain the data collected at each site (UT and BU) as independent data sets. Two sets of models will be developed, one trained to each site’s data, which will then be used to predict the other site’s data. All statistical tests of model predictions will be performed using data from a completely independent sample. This ensures that findings are not merely “statistically significant” in the sense of being improbable under a null hypothesis, but also demonstrates real-world predictive utility by directly showing that predictions based on one clinical sample are likely to generalize to other clinical samples of the same population. We refer to the site used to fit models as the “train site” and the site used to validate models as the “test site”.

Hypothesis testing will be based on a series of model comparisons. All models will be fit to the train site and used to predict the probability of treatment response at the test site. These probabilities will be used to generate an ROC curve for each model’s ability to discriminate treatment responders from non-responders. Significant differences between curves will be evaluated using DeLong’s test for two correlated ROC curves as implemented by the R package “pROC”. The following model comparisons will be performed:

1. All candidate predictors vs. excluding CO_2_/VH reactivity measures. This comparison addresses the question: do the novel measures of CO_2_/VH reactivity add predictive value beyond traditional—and easier to collect—measures like diagnosis or symptom severity? We hypothesize that the addition of the reactivity measures will significantly improve model performance. If CO_2_ reactivity is completely confounded with these competing predictors, then adding it to the model will not result in any predictive gains; CO_2_ reactivity can only improve model predictions if it provides unique information that is not captured by “confounders”.
2. CO_2_ vs. VH reactivity. Finally, if the above model demonstrates the value of collecting CO_2_ reactivity measures, we will next assess does CO_2_ reactivity specifically predict treatment non-response (by revealing something about the underlying biology), or is it merely heightened sensitivity to the physiological sensations that are common to both CO_2_ inhalation and hyperventilation? Qualitatively, if VH reactivity has no predictive utility, then the statistical learning algorithm will have penalized these measures out of the model entirely by this point. However, the hypothesis of mechanistic specificity will be more formally tested by comparing a model that includes just CO_2_ reactivity measures to one that includes just VH reactivity measures. Note that the ability to perform a head-to-head comparison like this is one of the advantages of comparing ROC curves in an independent test set; the models do not need to be nested as they would when performing likelihood ratio tests on model fits.

Two strengths of this validation strategy should be emphasized. First, the significance of the biomarkers will be determined by their ability to outperform self-report measures, not just that their coefficients are significantly different from zero. The former speaks to their real-world utility; the latter does not. Second, the biomarkers are being compared on their ability to predict new data, not just their ability to fit the same data. This design/analytic feature helps ensure that we do not propagate failure-to-replicate issues. If we do not replicate a simplified model across sites and our hypotheses are not supported by the data (and there is not support for an alternative model with adequate sensitivity and specificity), we will recommend to the field that reactivity to CO_2_ and/or VH challenge (and the confounder variables we assessed) should not be used for determining patients’ suitability for EBT. Our use of a replicated assessment and a large sample helps instill confidence in these recommendations.

#### Translating anticipated ROC gains into clinical impact

The comparison of the ROC model curves is a framework for hypothesis testing, but the model will ultimately need to be couched in other terms besides an improvement in the ROC to convince clinicians of its practical utility, which depends not only on the improved performance of the model, but also clinical judgment about the optimal tradeoff between sensitivity and specificity: one can always boost the gain in the correct number of non-responders at the cost of increasing the number of falsely identified non-responders, and individual clinicians and patient circumstances may dictate different cut points. Thus, a metric like ROC that captures the overall tradeoff between specificity and sensitivity across all possible decision thresholds is the best way to frame our minimal objective, keeping in mind that this is a conservative expectation; larger predictive gains are possible.

However, assuming the minimal gains in ROC performance that we used to determine sample size requirements, we can offer a working example for one potential cutoff: if we were to require greater than an 80% predicted probability of non-response to forego a trial of EBT, then, out of 5000 patients entering treatment, the standard baseline model would only identify 4 true non-responders and falsely identify 1 responder as a likely non-responder. In contrast, the addition of CO_2_ reactivity measures would identify 262 true non-responders while misidentifying 26 responders as non-responders. This would spare > 10% of non-responders from an unsuccessful trial of EBT vs. < 0.2% under the baseline model, while incorrectly excluding only 1% of would-be responders. The biomarker assessment burden in our protocol is a single session, balanced against 12 one-hour sessions of treatment. The ability to spare 1/10 non-responding patients from the burden of unsuccessful treatment, while only negatively impacting 1/100 responding patients, justifies the burden of a single assessment session, in our opinion.

#### Deriving treatment recommendation heuristics

The final data product is to translate the above models into algorithmic recommendations to determine whether a patient is very unlikely (*e.g.,* < 30% chance) to respond to EBT, which could support the recommendation of interventions that do not rely on fear extinction (e.g., other psychosocial interventions, pharmacological interventions). Although we chose a statistical learning algorithm that avoids “black box” predictions, obtaining a probability of non-response will still require inputting a number of measurements into something like a web-based calculator, which we will disseminate if the models demonstrate good predictive value. But clinicians might be more likely to adopt a treatment recommendation for a patient if they are able to derive it from decision rules (e.g., if a patient maintains a latency less than 90-s between CO_2_ inhalations) based on the measurements that are easiest to acquire. We will attempt to derive such decision rules by applying a recursive partitioning algorithm to the data (similar to the approach used to assist hospital pharmacy staff in identifying patients at risk of medication errors)[134] using only easy-to-obtain measures. We will then use the same model comparison strategy outlined above to compare the predictive performance of the decision rules to both a no-information model and the best models derived from elastic-net regression. If the simplified heuristic model offers significant gains over no information and is not significantly worse than the best models, then this will provide the ideal mechanism for translating the knowledge gained from this study to clinical practice.

#### Statistical power

We ran 5,000 computer simulations of our study design under a wide range of total sample sizes. In brief, a different multivariate distribution was defined for each of the following five diagnostic categories: (1) PD(with or without agoraphobia), (2) SAD, (3) GAD, (4) OCD, and (5) PTSD. Data were simulated to match the disorder-specific response rates (PD = 0.53, SAD = 0.45, GAD = 0.47, OCD = 0.43, PTSD = 0.59) reported in a meta-analysis of 87 studies [135]. The specified *N* for a given simulation was divided equally across the 2 sites and 5 disorder groupings within sites, and random samples of size *N*/10 were drawn for each disorder group for each site, one of which was arbitrarily labeled “train” and the other “test”. Just as we specified in our analytic plan, elastic-net logistic regression models were fit to the train site, and the optimal penalty mix (parameter alpha) and magnitude (parameter lambda) were selected using the average of 10 repeats of a tenfold cross-validation procedure. We then used this model to predict the response at the test site. These predictions (which are individual probabilities of response) and the actual response values were then used to generate ROC curves for the different models to be compared. The control model, which includes only “traditional” self-report measures, was assumed to have a true AUC of 0.63, and the experimental model, which adds CO_2_ and hyperventilation reactivity measures, was simulated to have a true AUC of 0.73. These values and their differences correspond to our minimal effect size of interest.

**Fig. 1:**
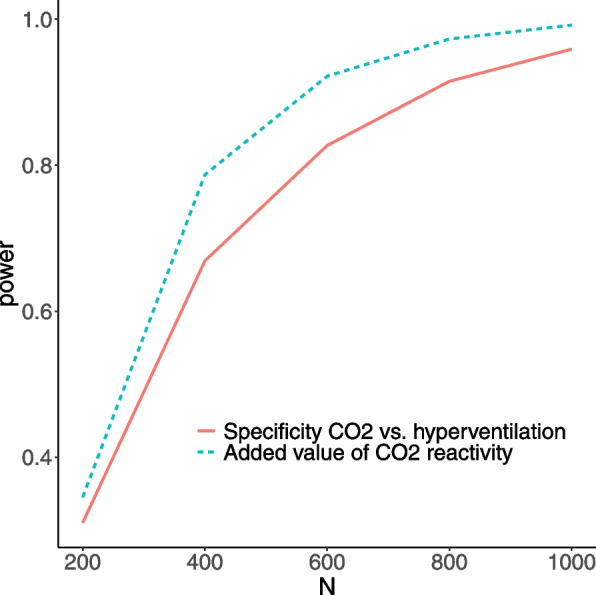
Power curve

Then, for each of the simulated data sets, area under the ROC curve (AUC) was compared using DeLong’s test for two correlated ROC curves, as implemented by the `roc.test` function in the “pROC” package in R, with the directional hypothesis that the experimental model has a greater AUC than the control model. At a total sample size of 600 (300 train site/300 test site), 92.2% of simulations found a significant (*p* < 0.05) difference in model performance. Applying the same approach to the comparison of models with only CO_2_ reactivity (no hyperventilation measures) vs. models with only hyperventilation reactivity (no CO_2_ measures), 82.7% of simulations found a significant (*p* < 0.05) superiority of CO_2_ over hyperventilation. The likely reason for this ~ 10% drop in power is that we modeled CO_2_ and hyperventilation reactivity measures as likely to be correlated (*r* = 0.03—0.25) such that hyperventilation would have a smaller, spurious relationship with treatment outcome by proxy. While this makes the mechanistic specificity of CO_2_ reactivity more difficult to detect than if we had assumed independence between CO_2_ and hyperventilation reactivity, these simulations show that we are reasonably well powered to detect the mechanistically specific predictive value of CO_2_ even under this noisy condition. Power curves for these model comparisons at all simulated sample sizes are shown in Fig. 1.

## Discussion

Anxiety-, obsessive–compulsive, and trauma- and stressor-related disorders reflect a significant public health problem. This study is designed to evaluate the predictive power of a novel biomarker based on a CO_2_ challenge, thus addressing the central question “can this easy-to-administer assay aid clinicians in deciding whether or not to initiate exposure-based therapy?”.

We hypothesize that the subjective and behavioral indices of CO_2_ reactivity will be the dominant predictors of EBT non-response. We do, however, include a set of competing predictors in our model. Hence, if our hypotheses are not supported and/or psychophysiological measures emerge as important predictors, we will still be able to deliver to the field an accounting of the variables/model that best predicts treatment failure. In essence, while our preliminary data provided a good basis to move forward, the work proposed in this application would comprehensively address possible confounders in a novel way. Ultimately, the final application of our work will be to recommend a specific assessment procedure and a specific go/no go assessment to guide clinical decisions for initiating exposure-based treatment vs. pursuing a different alternative.

Support for the study hypothesis that patients with elevated CO_2_ reactivity will not do well with EBT would justify the consideration to move away from this treatment modality altogether and instead select an alternative (e.g., psychosocial or pharmacological) modality for these patients, if indeed the biomarker is specific to EBT vs. an alternative treatment. As discussed, we have adopted a stage model approach to biomarker validation; targeting reliability of prediction in this study, with treatment specificity of prediction relegated to future investigations (i.e., we do not believe it is a cost-effective strategy to do both in the same initial trial). Moreover, we have considered a number of comparison conditions for a future study in the context of a randomized trial of two or more treatment arms, evaluating both alternative treatments and rescue strategies (i.e., a strategy that effectively recalibrates the mechanism that underlies the biomarker − EBT non-response relation), but all of these design decisions would be clearly informed by the results of the investigation proposed here (e.g., identification of the pre-randomization stratification point for CO_2_ reactivity). Finally, should the findings from the proposed study be consistent with the hypotheses, we will work with Hans Rudolph Inc. to develop accessible technology for assessing the relevant CO_2_ reactivity parameters thus aiding dissemination efforts.

## Data Availability

In line with NIMH guidance, we will share de-identified data derived from this study via the NIMH Data Archive (NDA; https://data-archive.nimh.nih.gov/), along with supporting documentation to enable efficient and appropriate use of the data. Data will be available under collection #4334. We agree that data will be deposited and made available through NDA, and that these data will be shared with investigators working at an institution with a Federal Wide Assurance (FWA) and could be used for secondary study purposes. All submitted data (both descriptive/raw and analyzed data) will be made available for access by qualified members of the research community according to the provisions defined in the NIMH Data Repositories Data Access Agreement and Use Certification. We agree to deposit and maintain the study data and secondary analysis of data (if any) at NDA. The repository has data access policies and procedures consistent with NIH data sharing policies. Descriptive/raw data will be shared on a semi-annual basis (on or before January 15 and July 15, beginning six months after the award budget period has begun). Analyzed data will be submitted prior to publication/public dissemination (whether the findings are positive or negative) using the NDA study feature that links data deposited in the NDA with publications/findings. We will include the entire analyzed dataset even if a publication only focuses on a specific aspect of the dataset. We will identify where the data will be available and how to access the data in any publications and presentations about these data, as well as acknowledge the repository and funding source. For each publication, a unique digital object identifier (DOI) will be created using the NDA Study feature, and this DOI will be included in the manuscript, linking the specific participants and data structures in the NDA that correspond to the analyses reported in each publication. The NDA has policies and procedures in place that will provide data access to qualified researchers, fully consistent with NIH data sharing policies and applicable laws and regulations.

## Abbreviations

AUC: Area under the receiver operating characteristic curve
AS: Anxiety sensitivity
ASI-3: Anxiety Sensitivity Index—3rd version
BEAQ: Brief Experiential Avoidance Questionnaire
BLA: Basolateral amygdala
CGI: Clinical Global Impression
CGI-S: Clinical Global Impression—Severity
CGI-I: Clinical Global Impression—Improvement
CNS: Central nervous system
CO_2_: Carbon dioxide
CSF: Cerebrospinal fluid
CSSR-S: Columbia-Suicide Severity Rating Scale
DOCS: Dimensional Obsessive–Compulsive Scale
DI: Distress intolerance
DTI: Distress Tolerance Index
EA: Experiential avoidance
EBT: Exposure-based therapy
GAD: Generalized anxiety disorder
GAD-7: GAD-7 scale
Hcrtr-1: Hypocretin-1
IE: Independent evaluator
IL: Infralimbic cortex
IU: Intolerance of uncertainty
IUS: Intolerance of Uncertainty Scale
LH: Lateral hypothalamus
O_2_: Oxygen
OASIS: Overall Anxiety Severity and Impairment Scale
OCD: Obsessive–compulsive disorder
PCL-5: Posttraumatic Stress Disorder Checklist for DSM-5
PD: Panic disorder
PDSS-SR: Panic Disorder Severity Scale—Self-Report
PHQ-9: Patient Health Questionnaire—9-item version
PTSD: Posttraumatic stress disorder
ROC: Receiver operating characteristic
SAD: Social anxiety disorder
SCID-5: Structured Clinical Interview for DSM-5
SPIN: Social Phobia Inventory
SBAF: Safety Behavior Assessment Form
VH: Voluntary hyperventilation
